# Proteomic ageing clocks: methods, mechanisms and the muscle connection

**DOI:** 10.1186/s13059-026-04220-w

**Published:** 2026-07-30

**Authors:** Elizabeth G. Reisman, Macsue Jacques, Robin Grolaux, Vadim N. Gladyshev, Nir Eynon

**Affiliations:** 1 Australian Regenerative Medicine Institute, Faculty of Medicine, Nursing and Health Sciences, Monash University, Clayton, VIC 3800 Australia; 2 Division of Genetics, Department of Medicine, Brigham and Women’s Hospital, Harvard Medical School, Boston, MA 02115 USA

**Keywords:** Proteomic clocks, Ageing biomarkers, Multi-omics, Skeletal muscle

## Abstract

Proteomic ageing clocks are emerging tools that estimate biological age from protein abundance patterns. Most models are developed using blood capturing inflammatory, metabolic, and extracellular proteins, including secreted signalling factors. Large-scale plasma studies now predict chronological age, disease risk and mortality, but muscle-related models remain indirect, relying on organ-enriched circulating proteins rather than biopsy-derived skeletal muscle clocks. We review current proteomic clock frameworks, computational approaches, platform considerations and validation strategies, highlighting the potential of muscle-based clocks to capture tissue and subcellular-specific ageing processes. We discuss future integration of spatial, single-cell, and multi-omic data to enhance mechanistic insight and translational relevance.

## Introduction

Ageing is a universal yet heterogeneous process, driven by the accumulation of molecular and cellular damage that leads to functional decline, frailty, increased disease susceptibility, and ultimately death [1]. Chronological age alone fails to capture this biological complexity, as individuals of the same chronological age often display markedly different physiological capacities and health outcomes. This divergence has fuelled growing interest in developing biologically relevant ageing clocks, quantitative models that estimate an individual’s “biological age” from molecular data [2, 3]. Such models aim to provide a more accurate reflection of physiological ageing and predict morbidity, mortality, and response to interventions with greater precision than chronological age alone.

The most widely adopted ageing clocks were built on DNA-methylation (DNAm) patterns, which change predictably with age across tissues [4, 5]. These clocks, including those by Horvath and Hannum [4, 5], established the concept of biological age and its links to disease risk and longevity [6]. However, while first-generation DNAm clocks are trained to predict chronological age and capture stable, cumulative signatures of ageing, they provide limited insight into dynamic physiological processes or functional status. Moreover, many epigenetic clocks are trained predominantly on blood-derived datasets and therefore have limited capacity to generalise across other tissues, where their predictive performance may decline [7]. Tissue-specific DNAm clocks have also been developed, including the skeletal muscle-specific Muscle Epigenetic Age Test (MEAT) and MEAT 2.0 [8, 9]. Trained directly on human muscle biopsies, these clocks outperform pan-tissue models and capture muscle-relevant ageing pathways [10]. Similar tissue-resolved epigenetic clocks have been developed, further illustrating how tissue context shapes molecular ageing signatures [11]. However, methylation-based models offer limited mechanistic insight and modest responsiveness to short-term interventions [10, 12], highlighting the need for additional molecular layers, particularly proteomics to capture functional aspects of ageing.

**Fig. 1:**
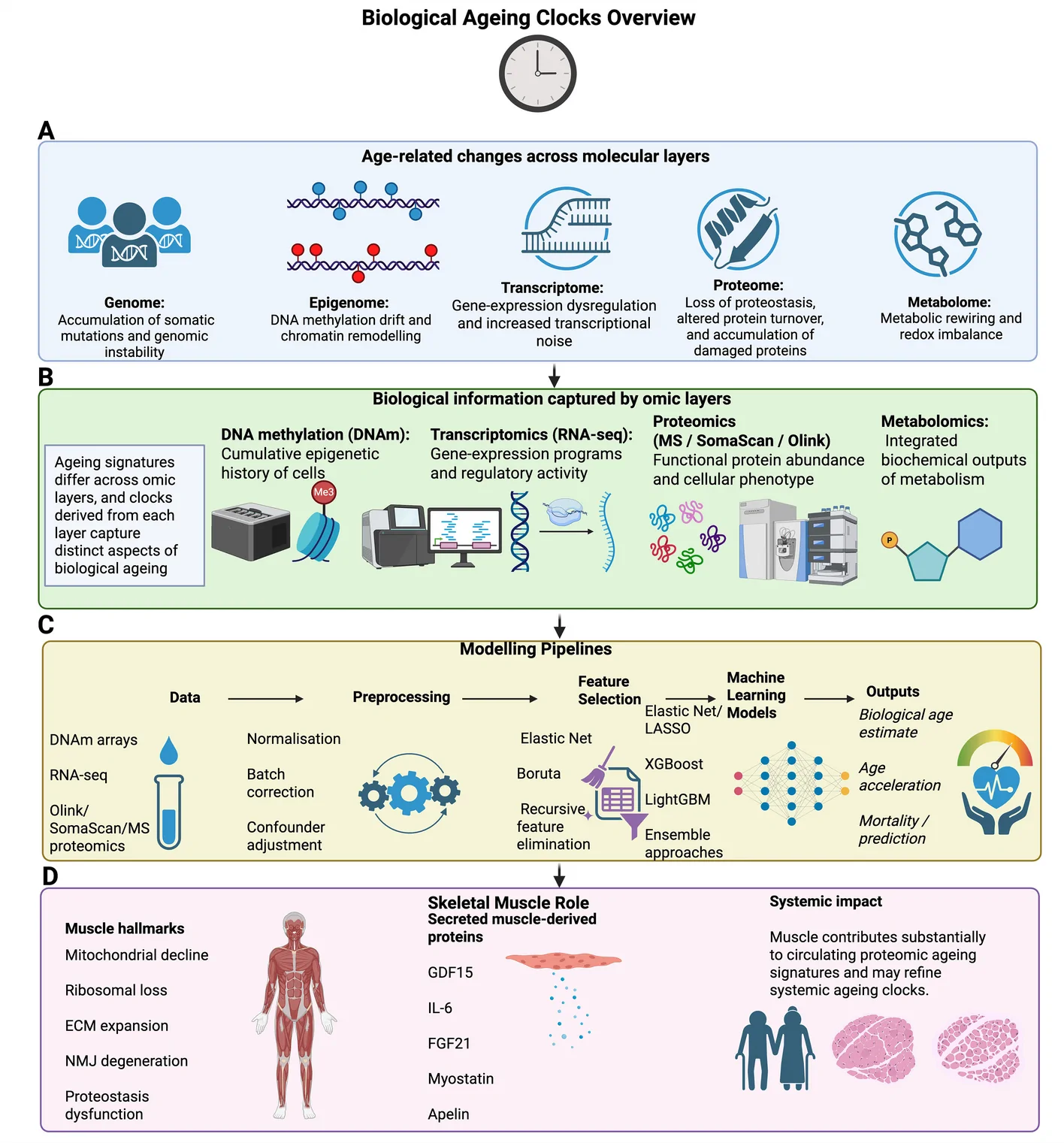
Biological ageing clocks integrate information across multiple molecular layers to estimate biological age. **A** Molecular substrates contributing to ageing include the genome, epigenome, transcriptome, proteome, and metabolome, each capturing changes over distinct temporal scales. **B** Each omic layer captures complementary aspects of biological ageing alongside the technologies used to measure them: DNA methylation (DNAm arrays) reflects cumulative epigenetic history; transcriptomics (RNA-seq) captures gene-expression programmes and regulatory activity; proteomics (e.g., mass spectrometry, SomaScan, Olink) reflects functional protein abundance and cellular phenotype; and metabolomics captures integrated biochemical outputs of metabolism. **C** Generalised modelling pipeline for ageing clocks, including data acquisition, preprocessing (normalisation, batch correction, confounder adjustment), feature selection (e.g., Elastic Net, Boruta), and machine-learning modelling (e.g., Elastic Net, XGBoost, LightGBM, ensemble approaches), generating outputs such as biological age, age acceleration, and risk prediction. **D** Skeletal muscle may contribute to systemic proteomic ageing signatures through key hallmarks, including mitochondrial dysfunction, ribosomal decline, extracellular-matrix remodelling, neuromuscular degeneration, and impaired proteostasis. Muscle-derived circulating factors (e.g., GDF15, IL-6, FGF21, myostatin, apelin) may contribute to systemic ageing signals and represent an opportunity to refine tissue-informed ageing models. Created with BioRender.com

Proteomic ageing clocks offer a more dynamic and mechanistically interpretable alternative. They model protein-level signatures associated with age and age-related physiology, offering a readout capturing biological ageing at the functional effector layer [13]. Proteins integrate transcriptional, translational, post-translational, and degradative regulation, providing a systems-level readout of pathway activity that reflects inflammation, metabolism, extracellular-matrix (ECM) turnover, and cellular stress responses more directly than DNAm or transcriptomic markers [14] (Fig. 1). Protein abundance is modifiable through interventions such as exercise, nutrition, and pharmacological treatment, positioning proteomic clocks as potentially more responsive indicators of biological ageing dynamics [15–17].

High-throughput proteomic technologies, including aptamer-based (SomaLogic), proximity-extension (Olink), and mass-spectrometry (MS)-based platforms, have enabled the measurement of thousands of proteins in human plasma or tissues at scale [18, 19]. Leveraging these datasets, recent studies have developed proteomic ageing clocks capable of predicting chronological age, mortality, and disease risk across diverse populations [20, 21]. The 2024 Nature Medicine study [21] demonstrates this progress by constructing a proteomic ageing model from over 200 age-related proteins using gradient-boosting algorithms and validating it across multi-ancestry cohorts [21]. Notably, many proteins repeatedly selected across independent proteomic clocks, including GDF15, CHI3L1, MMP12, WFIKKN2, and FGF21, represent conserved endocrine or inflammatory regulators of ageing, supporting the presence of core circulating proteomic signature linked to systemic biological decline. However, differences in proteomic platforms, including aptamer–protein binding bias, proximity extension assay performance, and MS dynamic range, remain important considerations. Future progress will depend on coordinated cross-platform benchmarking, including analysis of shared reference samples, development of reference matrices, and computational harmonisation frameworks that enable translation of clock features across platforms without attenuating genuine biological variation.

Despite this promise, proteomic ageing clocks remain predominantly blood-based, offering a systemic rather than tissue-specific view of ageing. Yet ageing progresses heterogeneously across organs, with multiple studies using pan-tissue epigenetic clocks demonstrating that different tissues age at distinct rates relative to chronological age [5, 22–24]. Skeletal muscle represents one such tissue that undergoes pronounced and functionally significant decline [25–28]. Proteomic measurements provide a complementary perspective by directly quantifying the abundance of the proteins executing these pathways, thereby linking regulatory changes to functional cellular machinery. Convergence between muscle and circulating proteomic signatures suggests a possible muscle contribution to systemic ageing signals, but whether tissue-informed protein features refine systemic ageing clocks or improve prediction of functional decline remains to be tested in paired muscle-blood cohorts [25, 29]. In addition, muscle ageing involves processes such as neuromuscular junction degeneration, altered satellite-cell dynamics, and fibre-type remodelling, features that are not readily inferred from circulating biomarkers alone [30, 31].

This review synthesises current advances in proteomic ageing clocks, emphasising methodological frameworks, computational strategies, and validation approaches. It further highlights the untapped potential of skeletal-muscle proteomics and evaluates the conditions under which muscle-derived proteomic information may add value to systemic ageing models. We also outline how proteomic clocks could evolve from predictive tools towards actionable biomarkers that capture both the pace, tissue specificity and modifiability of human ageing.

## Ageing OMIC clock concepts and methodological frameworks

Ageing clocks are statistical and machine-learning models designed to estimate an individual’s biological age from molecular features that change over time. The fundamental premise is that certain biomolecular signatures, spanning the genome, transcriptome, proteome, and metabolome encode information about the cumulative impact of ageing processes on cellular and systemic function [21, 32, 33]. By training models to predict chronological age, first-generation ageing clocks extract latent molecular features that reflect aspects of biological deterioration. Although these clocks are not designed to predict clinical outcomes such as morbidity or mortality, deviations between predicted and chronological age (age acceleration) is associated with increased disease risk, mortality, and reduced resilience to physiological stress [6, 34]. In contrast, second and third generation clocks are often trained directly on health-related outcomes or composite phenotypes, including mortality risk, clinical biomarkers or pace of ageing [35–37]. This framework assumes that ageing leaves a quantifiable molecular footprint, and that statistical learning can uncover patterns imperceptible to conventional phenotypic assessments. Importantly, age-related molecular drift accumulates in a probabilistic and multi-dimensional manner, meaning that many clock models rely on capturing subtle but coordinated changes across large numbers of molecular features rather than single biomarkers. Complementary frameworks have also been proposed, including stochastic and entropic clocks [38, 39], which quantify age-related increases in molecular variability or disorder rather than directional shifts in mean abundance.

Each omic layer captures distinct dimensions of ageing. DNA methylation reflects long-term epigenetic changes, including both stochastic epigenetic drift and directional alterations at specific genomic loci, such as age-associated differentially methylated positions enriched at polycomb repressive complex 2 (PRC2) target sites [40]. Transcriptomics capture intermediate regulatory states, and metabolomic and proteomic clocks provide readouts closer to functional physiology (Fig. 1). While DNAm clocks excel at estimating cumulative biological ageing, they respond slowly to environmental or therapeutic perturbations [13, 41]. In contrast, proteomic clocks reflect active signalling, inflammation, endocrine function, and metabolic state, and may shift more rapidly in response to interventions. These distinctions underscore that ageing clocks are not interchangeable, but rather complementary tools that capture ageing across different temporal and biological scales.

*Feature selection is a critical step in clock construction*. In proteomic clocks, algorithms such as Boruta or recursive feature elimination identify minimal protein sets with high predictive power [42]. Ensemble methods like Light Gradient Boosting Machine (LightGBM) and Elastic Net regression are frequently used to balance predictive performance with model interpretability [43]. More broadly, an important conceptual limitation of first-generation ageing clocks is that models optimised solely to predict chronological age preferentially capture molecular features that track the passage of time rather than biological ageing. This has motivated the development of second- and third-generation clocks that incorporate phenotypic endpoints such as morbidity, frailty, physical function, or mortality risk. For example, Higgins-Chen et al. introduced principal component (PC)-based epigenetic clocks that reduce technical noise and improve reproducibility across repeated measurements, making them better suited for longitudinal studies and clinical trials [44]. These developments reflect a broader shift towards biological-risk clocks that prioritise healthspan, resilience, and disease susceptibility as key dimensions of ageing. This reorientation aligns ageing-clock development more closely with geroscience priorities to identify predictive biomarkers that could ultimately be deployed in clinical settings.

Another methodological consideration is *managing confounders and variance sources inherent to omics-scale data*. Technical factors including batch effects, assay drift, protein degradation, and sample handling can introduce noise that obscures ageing signals. Importantly, ageing-clock development must distinguish between statistical variation arising from measurement noise and true biological variation reflecting ageing processes, as failure to separate these sources can reduce model reliability and reproducibility [45]. Biological confounders such as sex, ancestry, medication use, BMI, and inflammation also contribute systematic variance that must be modelled explicitly to avoid biased age estimates. Robust ageing clocks therefore rely on stringent data pre-processing, normalisation methods, and cross-cohort calibration to ensure that predictive signals reflect biological ageing rather than dataset-specific artefacts.

*Validation frameworks are equally important*. Chronological-age prediction performance is typically assessed using correlation coefficients (r), explained variance (R^2^), or mean absolute error (MAE). However, biological relevance is better captured by evaluating associations with mortality, frailty, functional decline, and disease incidence. These outcomes should not be conflated: a clock can predict chronological age accurately yet have limited association with health outcomes, while a model trained on morbidity or mortality risk may be clinically informative despite weaker chronological-age prediction. External validation in independent cohorts particularly across ancestries is essential for assessing generalisability. Longitudinal data, though still limited for proteomics, offer the opportunity to quantify rate of ageing through repeated measures, providing a trajectory-based dimension that single-timepoint clocks cannot capture. For example, DNAm models such as DunedinPACE estimate the pace of biological ageing using longitudinal phenotypic data, illustrating how repeated-measures designs can capture ageing dynamics [36].

Finally, *the computational landscape of ageing clocks continues to evolve*. While earlier clocks employed regularised linear models such as elastic net regression [5], contemporary approaches increasingly leverage non-linear methods, including gradient boosting and random forests, to capture complex ageing signals [46, 47]. These approaches can improve predictive performance but often reduce interpretability, particularly in higher-complexity models such as deep learning, which also remain limited by dataset size in most omics studies. As a result, interpretable machine-learning frameworks, including SHAP values and feature-attribution methods [48], are becoming central for uncovering the biological pathways underlying clock predictions, enhancing both mechanistic insight and translational potential. In summary, a strong proteomic ageing clock should combine transparent preprocessing, appropriate confounder control, external validation, clearly reported chronological-age and outcome-prediction metrics, and biological interpretation that distinguishes predictive association from causal mechanism.

## Proteomic ageing clocks: from blood to tissue

**Fig. 2:**
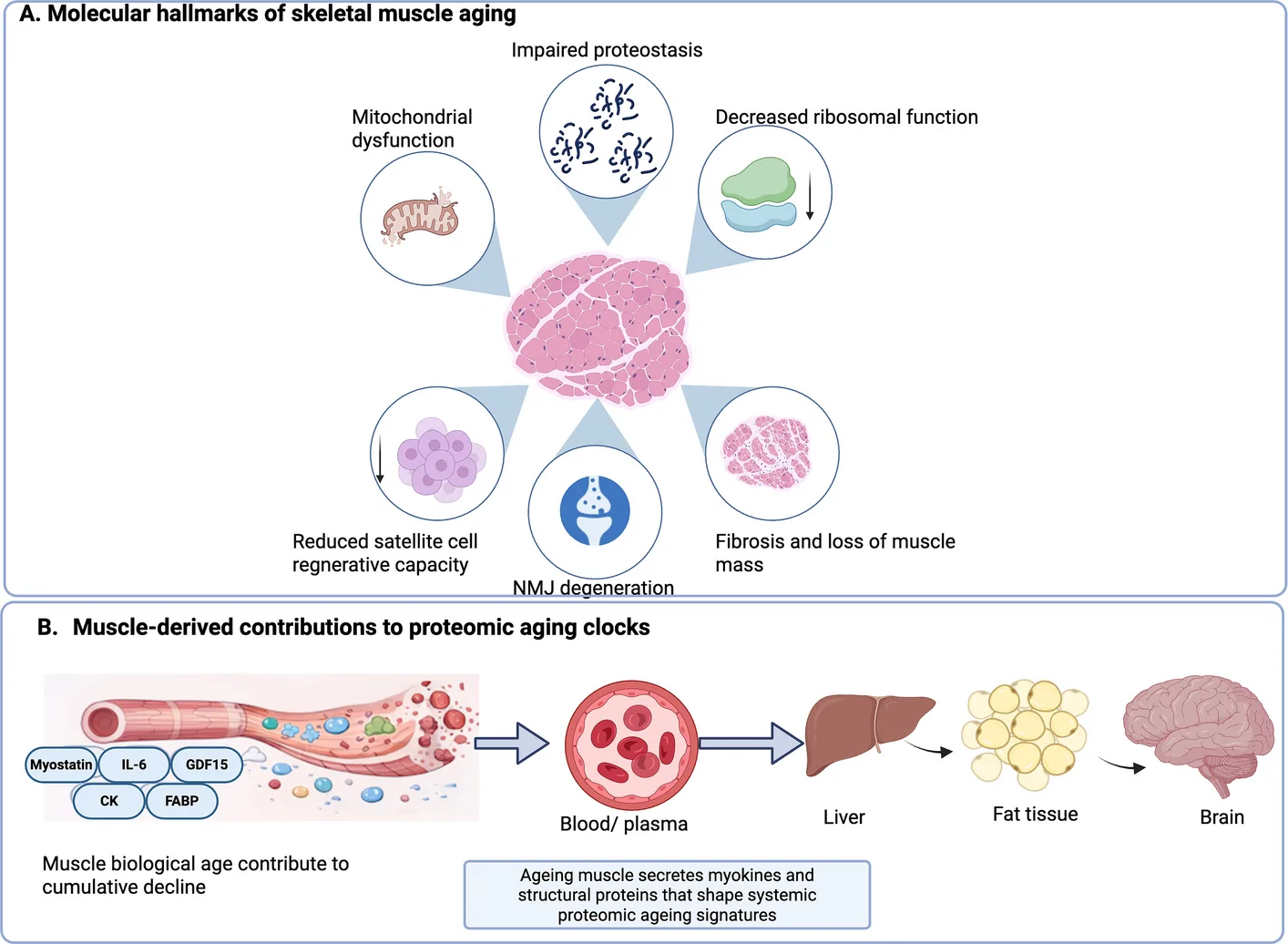
Rationale for a skeletal-muscle–derived proteomic ageing clock. **A** Ageing skeletal muscle exhibits hallmark features including mitochondrial dysfunction, impaired proteostasis, ribosomal loss, extracellular-matrix expansion and fibrosis, neuromuscular junction deterioration, and reduced satellite cell regenerative capacity. **B** Ageing muscle secretes a range of proteins and myokines including GDF15, IL-6, FGF21, myostatin, collagen fragments, FABP3, and CK into the circulation. These signals may arise through regulated secretion, extracellular vesicle release, tissue turnover, leakage or remodelling in other tissues such as blood, liver, fat and brain. This may shape plasma proteomic signatures that contribute to systemic ageing clocks. Created with BioRender.com

The proteome provides a closer-to-function readout of ageing by capturing cumulative changes in protein abundance, post-translational modification, and secretion across tissues. Early proteomic ageing clocks focused on plasma, where accessible circulating proteins reflect systemic physiological states [49, 50]. Initial studies using targeted mass-spectrometry identified individual proteins such as Growth Differentiation Factor 15 (GDF15) [51] and Matrix Metalloproteinase (MMPs) [52] as strong correlates of age. The advent of high-throughput affinity platforms SomaScan and Olink enabled models incorporating thousands of features, transforming the field [15, 53]. These platforms revealed that the circulating proteome undergoes non-linear age-related shifts across the lifespan [54], reflecting coordinated transitions in immune function, metabolism, and inter-organ communication (Fig. 2).

Although circulating proteomic signatures capture systemic ageing signals, a proportion of plasma proteins originate from specific tissues. Recent analyses suggest that approximately 25–30% of tissue-enriched proteins, including those derived from skeletal muscle, can be detected in plasma [55]. While this overlap indicates that circulating proteomes partially reflect tissue biology, plasma-based measurements integrate signals from multiple organs and therefore cannot resolve tissue-specific ageing trajectories. Recent efforts have attempted to infer organ-specific biological age from blood-based proteomic signatures, linking circulating protein patterns to dysfunction in tissues such as the liver, heart, and brain [56]. However, these approaches remain indirect proxies of tissue molecular states. Direct tissue-resolved proteomic clocks may provide a complementary strategy by quantifying ageing processes within specific organs rather than inferring them from circulating biomarkers. Many age-associated plasma proteins are linked to pathways such as inflammation, ECM remodelling, and metabolic regulation, processes that also characterise ageing in skeletal muscle and other tissues [57, 58]. This overlap highlights the connection between circulating and tissue ageing biology, while underscoring the need for tissue-resolved proteomic approaches capable of directly capturing the molecular processes underlying functional decline. For skeletal muscle, this distinction is central where circulating muscle-associated proteins should not automatically be interpreted as direct readouts of myofibre ageing. Paired muscle–plasma datasets are needed to determine whether these proteins track biopsy-derived muscle proteomic ageing, functional phenotypes or broader systemic inflammatory states.

**Fig. 3:**
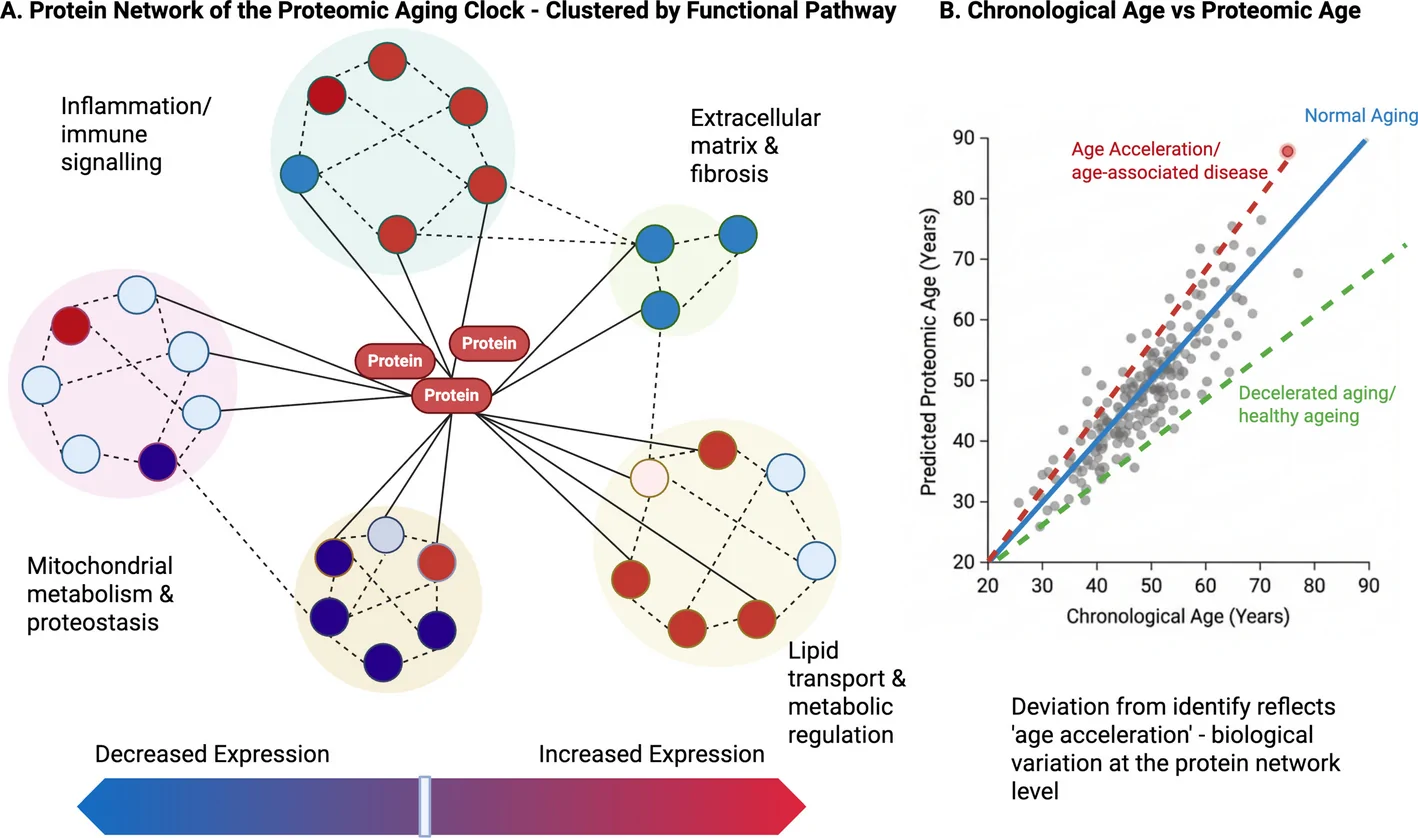
Proteomic ageing clocks encode conserved functional protein networks. **A** Proteomic ageing clocks are composed of recurrent sets of age-associated proteins that form interconnected networks rather than acting as isolated biomarkers. Across independent cohorts and platforms, proteins selected into these models cluster into coordinated functional modules, reflecting shared biological pathways and system-level organisation. Many of these proteins are circulating and derive from multiple tissues, highlighting how tissue-level molecular changes may contribute to systemic proteomic ageing signatures. This network-based architecture supports biological interpretation, but these proteins should be interpreted as predictive or age-associated markers unless causal evidence is available. **B** The resulting models generate estimates of biological age, with deviations from chronological age reflecting age acceleration or deceleration. Created with BioRender.com

A key milestone for proteomic clocks was a study by Argentieri et al. [21], which constructed a 204-protein LightGBM model across 51,408 individuals from multiple ancestries. The clock predicted chronological age with high accuracy (MAE ≈ 3.4 years) and deviations between predicted and chronological age (proteomic age acceleration) were significantly associated with mortality and multiple disease outcomes. The top predictors represented inflammatory, ECM, and metabolic pathways, processes that are also prominent features of ageing in skeletal muscle and other tissues [59]. Other large proteomic clocks have reported similar findings, consistently identifying proteins such as CHI3L1, PI3, WFIKKN2, MMP12, CTSZ, and FGF21 as highly age-associated across cohorts and platforms. The recurrent appearance of these proteins suggests a conserved core signature of systemic ageing centred around inflammageing, innate immunity, ECM remodelling, and metabolic dysregulation [50] (Fig. 3). Notably, several of these proteins are known myokines or ECM regulators produced by muscle, providing tangible examples of how some tissue-level ageing processes are reflected in circulating proteomic profiles.

Incorporating skeletal-muscle proteomic information into ageing-clock frameworks may improve biological interpretation of functional decline, but whether it improves prediction beyond plasma-based models remains to be tested in paired muscle, blood and phenotype datasets. In a multi-compartment ageing analysis, skeletal-muscle age-acceleration scores were associated with usual-pace walking speed (Spearman r = 0.35, *p* = 0.016), providing initial evidence that muscle proteomic ageing signatures relate to physical function [58]. Retained muscle-associated features in this study included FLNC, MYBPH, SSPN, OXCT1, HSPB1, BCL2L13, FHL3 and MUSTN1, linking the muscle signature to structural integrity, metabolism and stress-response pathways [59]. However, the extent to which muscle-derived or muscle-responsive proteins contribute to circulating proteomic clocks remains inferred rather than directly demonstrated.

Insights from epigenetic clock research provide a useful conceptual framework for addressing these challenges in proteomic ageing clocks. Beyond skeletal muscle, other tissues including adipose tissue, liver, and immune compartments exhibit distinct molecular trajectories of ageing, each contributing partially overlapping but non-identical signals to the circulating proteome. This concept has been formalised in large-scale multi-tissue DNAm atlases, which demonstrate that ageing reflects both conserved cross-tissue programmes and tissue-specific regulatory modules. A recent example is the DNA Methylation Ageing Atlas Across 17 Human Tissues [23] which integrated DNAm profiles across a wide range of human tissues and identified both shared and tissue-enriched ageing signatures. This atlas reported conserved age-associated methylation changes in genes such as PCDHGA1, MEST, HDAC4, and multiple HOX loci, alongside tissue-specific modules linked to metabolism, inflammation, and extracellular remodelling. These findings provide testable targets for proteomic follow-up, raising the question of whether conserved DNAm ageing drivers are mirrored by coordinated, multi-tissue protein-level changes and whether such proteins contribute to stable circulating ageing signals.

### Cell-type specific proteomic clocks

**Fig. 4:**
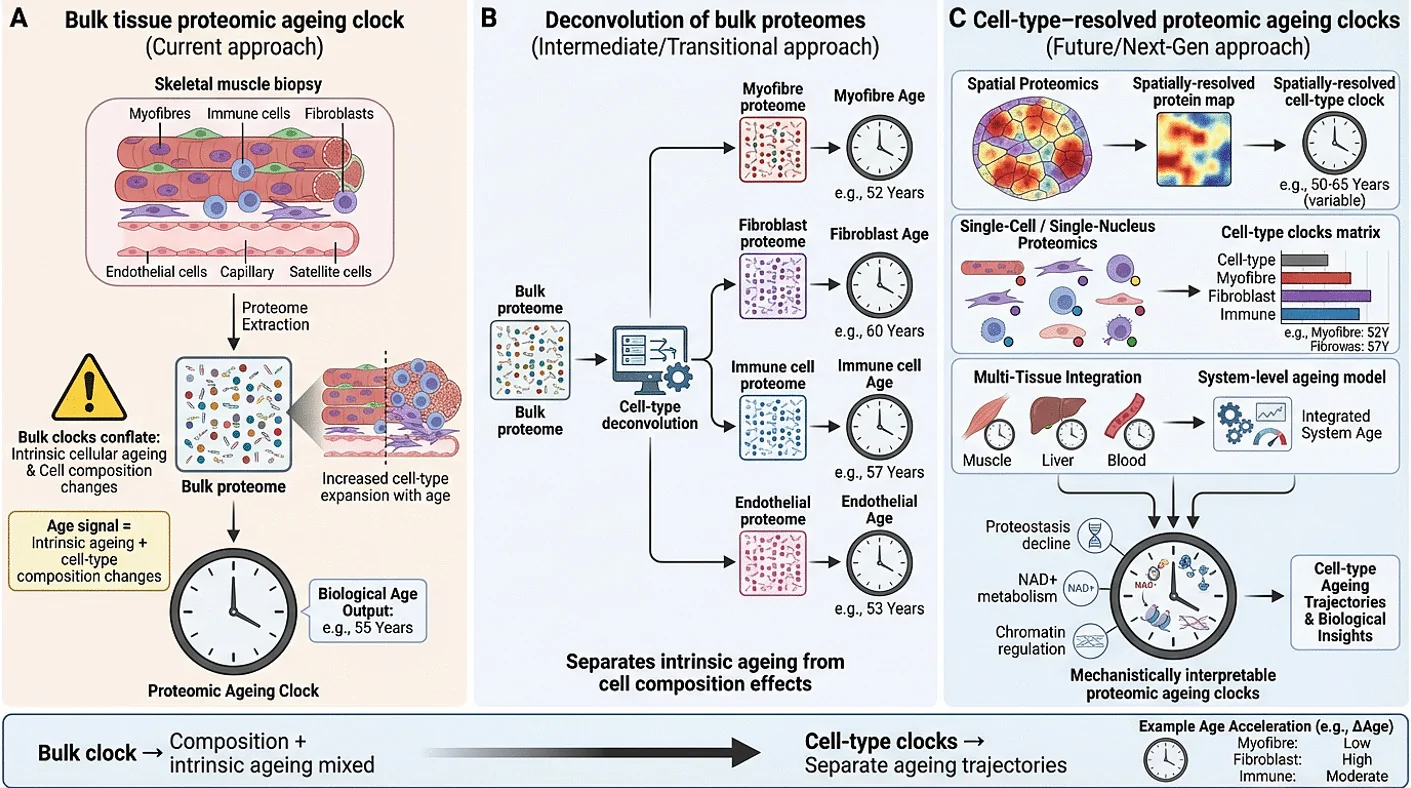
Conceptual framework for cell-type–resolved proteomic ageing clocks. **A** Current proteomic clocks are typically trained on bulk tissue or circulating proteomes, which integrate signals from multiple cell types and may conflate intrinsic molecular ageing with age-related changes in cellular composition. **B** Computational deconvolution of bulk proteomic datasets can estimate cell-type contributions and enable construction of lineage-specific ageing models. **C** Emerging technologies including spatial proteomics and single-cell proteomics will allow direct measurement of protein abundance within defined cellular populations, enabling cell-type–resolved proteomic ageing clocks and improving mechanistic interpretation of biological ageing across tissues. Created with figurelabs.ai

A major conceptual advance from epigenetic clock research is the recognition that bulk tissue clocks conflate intrinsic cellular ageing with age-related shifts in cell-type composition. This has motivated the development of cell-type specific epigenetic clocks [60], which estimate biological age within defined cellular populations and reveal clearer associations with disease risk and tissue dysfunction than bulk-tissue models (Fig. 4). For example, Tong et al. [60] introduced cell-type–resolved epigenetic clocks that demonstrated differential ageing rates across lineages and showed that a hepatocyte-specific clock better detects liver pathology-associated age acceleration than bulk-tissue clocks. More recently, systematic reviews have emphasised that explicitly modelling cellular heterogeneity using reference-based deconvolution, single-cell methylation data, or hybrid approaches is essential for improving interpretability, generalisability in ageing clocks [45]. Together, these studies demonstrate that disentangling intrinsic molecular ageing from age-related changes in cell-type composition is essential for accurate interpretation of ageing clocks. While most clocks remain primarily predictive, recent causality-enriched frameworks have begun to explore whether specific molecular features may represent candidate contributors to ageing processes rather than purely correlational markers [2].

Proteomic ageing clocks are now well positioned to adopt the same framework. In principle, cell-type–resolved proteomic clocks can be constructed by integrating bulk tissue proteomes with protein-based deconvolution, spatial proteomics, or emerging single-cell and single-nucleus proteomic technologies. Such approaches would allow separation of composition-driven ageing (e.g., immune or fibroblast enrichment) from intrinsic cellular proteostasis decline, while enabling direct testing of conserved ageing drivers identified in DNAm atlases [23]. By aligning tissue- and cell-type–specific proteomic ageing signatures with circulating protein profiles, future multi-tissue proteomic clocks could achieve greater mechanistic clarity, explicitly attribute signals to their tissue or cellular origin, and improve prediction of functional ageing phenotypes.

Despite their promise, single-cell and spatial proteomic approaches are not yet routine platforms for ageing-clock development. Relative to bulk tissue and plasma proteomics, they currently involve trade-offs in proteome depth, throughput, cost, sample-processing complexity and cohort scale, which limits their immediate use for population-level model training. Their near-term value is therefore likely to lie in resolving cell-type origins of ageing signals, nominating mechanisms, and validating tissue sources of proteins identified in larger bulk or circulating proteomic clocks [61–63].

## Skeletal muscle proteome: a window into biological ageing

Skeletal muscle is among the earliest tissues to exhibit age-related decline, including sarcopenia, mitochondrial dysfunction, and loss of proteostasis [1, 2]. Proteomic analyses reveal mitochondrial downregulation, ribosomal protein loss, and ECM expansion as key hallmarks of aged muscle [4, 25] which align closely with systemic proteomic ageing signatures in plasma (Fig. 2). Rather than reflecting isolated pathways, muscle ageing appears to involve coordinated disruption of energy production and protein homeostasis, characterised by impaired mitochondrial function, reduced translational capacity and loss of proteome integrity.

Ageing-related remodelling also extends beyond muscle fibres to the surrounding microenvironment. Dysregulation of fibro-adipogenic progenitors promotes ECM deposition and fibrosis, with increased abundance of collagens, laminins, and fibronectin, alongside satellite-cell dysfunction, indicating impaired regeneration [64]. These microenvironmental changes contribute to sarcopenia independent of muscle fibre atrophy, highlighting the multifaceted nature of muscular ageing. In parallel, proteomic evidence of neuromuscular junction (NMJ) disruption highlights a neurogenic component to muscle ageing, associated with fibre-type transitions and emerging as a key predictor of functional impairment in later life [65]. Together, these findings suggest that muscle ageing reflects coordinated alterations across structural, regenerative, and neuromuscular systems, with potential relevance for refining proteomic ageing clocks.

Age-associated changes in proteostasis-related proteins may also reflect impaired autophagy and lysosomal quality-control pathways, which are central to removal of damaged proteins, dysfunctional mitochondria and aggregated cellular material in skeletal muscle [26]. These processes intersect with anabolic and nutrient-sensing pathways already implicated in muscle ageing, including IGF-1/AKT/mTORC1 signalling, where age-related dysregulation may contribute to impaired protein synthesis, anabolic resistance and reduced maintenance of muscle proteome integrity. With ageing, reduced autophagy/mitophagy and altered chaperone-mediated autophagy may further contribute to mitochondrial dysfunction, myofibre atrophy and impaired adaptation to stress or exercise [66, 67]. Similarly, age-related increases in inflammatory and stress-response proteins may partly reflect accumulation of senescent cells within muscle fibres, satellite-cell niches or stromal compartments [26, 68]. Through the senescence-associated secretory phenotype, inflammatory cytokines, chemokines and ECM remodelling proteins may amplify local inflammation, fibrosis and regenerative impairment, meaning that such proteins may act both as markers of tissue damage and as contributors to progressive muscle dysfunction.

A key distinguishing feature of skeletal muscle is its plasticity. Exercise induces rapid and coordinated proteomic adaptations [69] and several exercise-responsive proteins overlap with plasma proteomic ageing signatures including circulating myokines and stress responsive proteins such as GDF15, FGF21, and IL-6 [51, 70]. This overlap supports a testable model in which muscle-derived or muscle-responsive signals contribute to circulating ageing biomarkers. Whether these exercise-responsive proteins alter proteomic biological age, and whether such changes originate primarily from muscle or broader systemic responses, remains unclear.

Integrating skeletal-muscle and circulating proteomic data may help determine the tissue origins of systemic ageing signatures; however, the contribution of muscle-derived or muscle-responsive proteins to plasma ageing clocks has not yet been directly established. Paired muscle and plasma datasets could identify age-related proteins influenced by muscle ageing, although longitudinal or mediation analyses are needed to determine whether these signals reflect muscle decline, immune dysregulation, metabolic dysfunction or broader systemic ageing. A muscle-focused proteomic clock would be expected to derive information from protein classes that are poorly represented or difficult to interpret in blood alone. These include sarcomeric and fibre-type proteins such as MYH7, MYH1/2, ACTN2, TNNT and TPM isoforms; mitochondrial oxidative-phosphorylation and tricarboxylic-acid-cycle proteins; ribosomal and translation-associated proteins; proteostasis machinery including chaperones and ubiquitin–proteasome components; ECM proteins such as collagens, laminins, fibronectin and periostin; and muscle-derived cytokines or myokines such as IL-6, GDF15, FGF21, myostatin and apelin. These features may reflect different biological processes: some mark intrinsic myofibre ageing, some capture fibre-type remodelling or denervation, and others indicate inflammation, fibrosis, regeneration or tissue turnover. Interpreting muscle-clock features in relation to specific hallmarks of muscle decline, rather than as generic age-associated proteins may therefore help clarify their biological meaning and potential clinical relevance. Taken together, skeletal muscle is a biologically relevant tissue for proteomic ageing research because of its direct contribution to mobility, metabolic health and age-related functional decline [26].

## Cross-clock methodological comparison and advantages of proteomic ageing clocks

Biological ageing clocks differ in their features of interest, computational frameworks, and predictive applications. Accordingly, clock performance should be interpreted in relation to the biological or clinical question each model is designed to answer, rather than by chronological-age prediction accuracy alone. DNAm clocks remain the most widely adopted predictor of chronological age (often r > 0.95) reflecting the ability of methylation to track cumulative processes [4–6]. In contrast, transcriptomic clocks capture dynamic biological processes such as immune activation and metabolic regulation, however, their performance is more context-dependent, and often tissue-specific. Early transcriptomic models showed strong within-cohort performance but limited transferability across datasets and cell compositions [71]. Subsequent models report moderate predictive accuracy (r ≈ 0.6–0.9) that varies by tissue and validation strategy [72–74]. Together, these findings indicate that transcriptomic clocks trade increased biological sensitivity for reduced cross-cohort robustness. Proteomic clocks generally show slightly lower chronological-age prediction performance than DNAm clocks, with reported correlations ranging from approximately r = 0.70–0.94. For example, the 204-protein ProtAge model reported a Pearson correlation of r = 0.94 and MAE of approximately 3.4 years for chronological age prediction, whereas the proteomic ageing clock (PAC) model reported a Spearman correlation of approximately 0.77 between proteomic age and chronological age [21, 49]. However, these metrics capture only one dimension of clock performance. A model with strong chronological-age prediction is not necessarily the best predictor of disease, mortality, functional decline or intervention responsiveness.

This distinction is particularly relevant for second and third-generation clocks which are often evaluated using outcome-specific metrics rather than age-prediction accuracy alone. ProtAge was associated with incidence of 18 major chronic diseases, multimorbidity and all-cause mortality, with each decile increase in proteomic age gap associated with an approximately 1.9-fold higher mortality risk [21]. Similarly, PAC was developed using all-cause mortality risk as a biological-age proxy and predicts mortality and several incident age-related diseases [49]. More recent Olink-based organ ageing models reported strong cross-cohort reproducibility (r = 0.98 and r = 0.93), and showed that accelerated organ ageing predicted disease onset, progression and mortality beyond clinical and genetic risk factors [50].

As summarised in Table 1, proteomic ageing clocks span a broad spectrum of study designs, from large-scale plasma-based models to emerging tissue-resolved approaches, and incorporate diverse proteomic platforms (e.g. SomaScan, Olink, MS-based proteomics) and modelling strategies. This diversity reflects both technical constraints and biological intent, but it also means that proteomic clocks should not be interpreted as equivalent measures of ageing across tissues. Large plasma-based models provide scalable biomarkers of systemic ageing and disease risk, whereas tissue-resolved approaches may capture more localised ageing processes that are less visible in circulation.

**Table 1:** Comparison of major proteomic ageing clocks across tissues, platforms, and modelling approaches

Study	Clock vs ageing biology	Tissue/sample	Cohort (n; age)	Proteomics technique	Model method	Generation	# proteins (measured → feature model)	Main findings
Ding et al., Cell (2025) [75]	Multi-tissue proteomic atlas & tissue-specific proteomic age clocks	13 human tissues including skeletal muscle from organ transplant donors	516 samples, ages 14–68	LC–MS/MS Orbitrap Astral	glmnet – elastic net model with LOESS correction	1^st^ gen tissue specific ageing clocks; + 2nd gen mortality-risk score	6,690 → 199 muscle feature proteins 1,732 → 39 plasma feature proteins	Identifies age inflection ~ 50y, early ageing in vasculature, proteostasis decline/amyloid features; produces organ/tissue clocks and “senoproteins” such as GAS6
Oh et al., Nature (2023) [76]	Plasma-derived organ-age from organ-enriched circulating proteins. Models trained on chronological age	Plasma organ-enrichment	5,676 adults across 5 cohorts; training cohort *n* = 1,398, mean age 75, range 27–104	SomaScan aptamer proteomics	Bagged ensemble LASSO age predictors	1^st^ gen organ-specific clocks; + mortality/disease-prediction evaluated downstream	4,979 → 4,778 (model inputs differ by organ-enriched subsets) → 856 organ enriched	Builds 11 organ clocks including muscle built off plasma proteome organ age gaps weakly–moderately correlated across organs; accelerated organ ageing predicts organ-specific disease risk and mortality
Wang et al., Nature Aging (2025) [50]	Organismal and ten plasma-derived organ clocks trained on chronological age	Plasma	UKB n = 46,673, CKB *n* = 3977, NHS *n* = 800	Olink Explore 3072 affinity proteomics	LightGBM	1^st^ gen organ age prediction	2916 → 240 proteins in organismal clock	Emphasises organ ageing quantification and prediction of disease/mortality and highlights neurodegenerative applications
Argentieri et al., Nature Medicine (2024) [21]	Chronological-age-trained plasma clock (ProtAge) with disease and mortality assessed as downstream validation	Plasma	Training in UKB, FinnGen, CKB; key age ranges include UKB ~ 40–70 (*n* = 45,441), FinnGen 20–80, (*n* = 1990) CKB 30–79, (*n* = 3977)	Olink Explore 3072 affinity proteomics	LightGBM	1^st^ gen plasma clock + extensive disease and mortality validation	2897 → 204 plasma proteins	ProtAge predicts mortality and incident age-related disease (18 major diseases) across populations; strong benchmark clock for “systemic” proteomic ageing. Key age and sex related proteins identified ELN, EDA2R, LTBP2, NEFL, CXCL17, SCARF2, CDCP1, GFAP, GDF15, PODXL2 and PTPRR
Goeminne et al., Cell Metabolism (2025) [77]	Plasma-based organ-specific proteomic ageing clocks; models biological ageing at the level of individual organ systems and links accelerated organ ageing to disease risk and mortality	Plasma	UKB; *n* = 44,952	Olink Explore 3072 affinity proteomics	glmnet – elastic net model with LASSO	Paired 1^st^ gen age clocks + 2^nd^ gen mortality-trained clock	2923 proteins → 2329 associated with age + 892 associated with mortality	Develops multiple organ-resolved proteomic ageing clocks. Demonstrates that accelerated ageing of specific organ systems predicts corresponding disease risk and all-cause mortality. Key markers GDF15 and NEFL
Lehallier et al., Nature Medicine (2019) [78]	Foundational plasma ageing proteome dynamics	Plasma	4,263 participants, 18–95 years	SomaScan aptamer proteomics	Differential expression-sliding window analysis (DE-SWAN)/trajectory clustering (nonlinear “waves”)	No predictive clock model	2,925 proteins	Identifies nonlinear “waves” of proteomic change (notably ~ 40 s, ~ 70 s, ~ 80 s) and pathway shifts across lifespan Key proteins (strong age association): SOST, ARFIP2, GDF15. (Also strong sex-associated proteins: CGA/FSHB, CGA/CGB, KLK3.)
Moaddel et al., Aging Cell (2025) [59]	Multi-omic ageing signatures	Plasma, urine and skeletal-muscle proteomics from Ubaida-Mohien et al.	22–92 years (*n* = 166 plasma), (*n* = 94 urine), muscle (*n* = 50)	Olink Explore 1536 (plasma/urine) TMT LC–MS/MS (muscle)	Elastic-net regularised regression with hierarchical cross-validation (eNetXplorer)	1^st^ gen ageing model	2,516 → 47 muscle feature proteins 1,432 → 73 plasma feature proteins 1055 → 75 urine feature proteins	Multi-compartment proteomic and metabolomic age predictors revealed concordant biological ageing deviations linked to inflammation, muscle mass and organ function. (e.g., FLNC, HSPB1, OXCT1, etc.) Key proteins in muscle signature (examples from retained features list): SSPN, PSAP, MYBPH, FLNC, OXCT1, HSPB1, BCL2L13, FHL3, MUSTN1. Overlap: MYBPH and SSPN also reported as age-associated in skeletal muscle proteomics by Ubaida‑Mohien (2019)
Ubaida-Mohien et al., eLife (2019) [25]	Deep ageing skeletal muscle proteome map (reference set for building many muscle-age signatures)	Skeletal muscle (biopsies)	60 participants, 20–87 years	TMT (tandem mass tag) LC–MS/MS proteomics	Differential expression/pathway analyses	No predictive clock model	4,380 proteins	Ageing human skeletal muscle shows reduced ribosomal and mitochondrial metabolic proteins and increased immune and proteostasis pathways Defines age-associated muscle proteome remodelling; key resource for “muscle aging biology” rather than prediction. Key skeletal‑muscle proteins highlighted: MYBPH, MFAP4, HLA‑DRB1, DOCK4 (under‑represented with age); SSPN, LIMA1, PLEC (within top age‑associated structural/sarcomeric cluster). Overlap: MYBPH and SSPN included in Moaddel (2025) muscle signature
Kuo et al., Aging Cell (2024) [49]	Proteomic ageing clock (PAC); systemic, not tissue-specific	Plasma	UKB, ~ 53,000 participants, 39–70 y	Olink Explore 3072 affinity proteomics	Lasso Cox regression–based proteomic ageing clock (PAC) followed by Gompertz mortality-age transformation	2^nd^ gen plasma ageing clock based on mortality associated proteins	2,923 → 128 plasma feature proteins for mortality-trained PAC	Proteomic age strongly predicts mortality and multiple age-related diseases beyond chronological age; represents a robust plasma-based proteomic ageing clock, not a skeletal muscle clock
Oh et al., Nature Medicine (2025) [79]	Plasma-derived organ-age models trained on chronological age	Plasma	UKB (*n* = 44,498) age 40–69	Olink Explore 3072 affinity proteomics	Bootstrap aggregated LASSO ageing models	1^st^ gen, organ-specific plasma PAC with longitudinal, disease and mortality validation	2916 proteins → 11 organ-specific age models → 128 muscle feature proteins	Organ-specific plasma proteomic age predicted disease and mortality, with brain ageing conferring Alzheimer’s risk comparable to APOE4
Tang et al., Nature Metabolism (2025) [80]	Age-associated proteomic remodelling/healthy ageing score	Serum	Longitudinal adult cohorts; *n* = 3796	Serum based MS-TripleTOF	Machine-learning–derived Proteomic Healthy Ageing Score (PHAS) based on random forest model	2^nd^ gen PHAS	438 serum proteins 86 longitudinally validated ageing-related proteins → 83-protein chronological-age model or 22-protein PHAS	Longitudinal serum proteomics identified ageing-associated proteins and a 22-protein proteomic healthy ageing score that predicts cardiometabolic disease risk and is modulated by gut microbiota
Menni et al., The Journals of Gerontology Series A (2015) [81]	Plasma proteomic signatures of chronological age	Plasma	202 female twins & 677 independent replication; mean ages ~ 65 & ~ 77 y	SomaScan aptamer proteomics	Linear regression & backward selection	Age-associated protein panel/ no-predictive clock model	1129 proteins → 11 replicated age-associated proteins → 4 plasma feature set proteins	Identified 11 plasma proteins (i.e. CHRDL1, CCDC80, PTN, ROR1, CST3, FSTL3, HAVCR2, IGFBP6, MMP12, TIMP1, and THBS4) that correlate with chronological age (replicated across cohorts), with a small panel reflecting age-related shifts linked to cardiovascular risk and developmental traits

*UKB UK Biobank, *CKB* China Kadoorie Biobank, *FinnGenn* Finnish health registry data, *NHS* Nurses’ Health Study, *LC-MS* Liquid Chromatography - Mass Spectrometry

This distinction is particularly important when considering the added value of skeletal muscle-based ageing clocks. Direct skeletal muscle proteomic clocks may provide complementary mechanistic value if they capture biopsy-specific ageing processes not recoverable from circulating proteins alone. At present, however, the evidence base comprises established muscle-ageing proteomic signatures and a small number of recently developed tissue-specific age models, rather than a mature field of externally validated skeletal-muscle-specific proteomic clocks. Therefore, the key unresolved question is whether direct skeletal muscle proteomics provides predictive or mechanistic information beyond plasma clocks and plasma-derived organ-age models.

An important shared feature across omic layers is the influence of tissue context. DNAm and transcriptomic clocks typically achieve optimal performance when derived from specific tissues or purified cell populations, such as whole blood leukocytes or tissue biopsies [7, 74,82]. Similarly, while most proteomic ageing clocks have been developed using circulating plasma proteins due to cohort size and scalability [5, 33] this comes at the cost of reduced tissue specificity. Circulating proteomes integrate signals from multiple organs, potentially obscuring cell- and tissue-specific ageing processes. Although tissue-resolved proteomic studies in human brain [83] and skeletal muscle [25] have identified robust age-associated protein signatures, these have rarely been translated into formal ageing clocks. Thus, tissue-resolved proteomic models may be most informative when the aim is to understand organ-, cell- or compartment-specific mechanisms of ageing, rather than to develop broadly scalable screening tools.

Although proteomic ageing clocks are frequently interpreted in an organ-specific context, most published infer tissue ageing indirectly rather than measuring it directly. As highlighted in Table 1, plasma-based organ clocks [50, 76, 77] rely on proteins annotated as organ-enriched using reference atlases. For example, muscle-associated ageing signals in plasma include proteins such as GDF15, COL6A3, ELN, TNNI2, FGF6, ADSSL1, PGM1, and BIN1 [5]. Importantly, many of these proteins are not classical myokines (i.e. actively secreted signalling molecules), but intracellular or structural proteins. Their detection in circulation likely reflects tissue turnover, remodelling, or cellular leakage rather than regulated secretion, underscoring that plasma-derived organ clocks represent inferred, rather than direct, measures of tissue ageing [21, 50,78].

In contrast to plasma-based models, direct skeletal muscle proteomic ageing signatures remain comparatively rare and have largely identified age-associated signatures rather than validated ageing clocks. A systematic review of human proteomic ageing studies identified skeletal muscle vastus lateralis as one of the most informative non-blood tissues, reporting 916 age-associated proteins, while earlier smaller muscle studies reported more limited age-associated protein sets [25, 29]. Deep muscle proteomics [25] identified age-associated declines in structural and mitochondrial proteins alongside increased immune and proteostasis-related pathways, while Moaddel et al. [59] incorporated skeletal muscle into a multi-compartment ageing framework, identifying retained muscle-specific features such as MYBPH, SSPN, FLNC, and OXCT1. More recently, Ding et al. [75] developed proteomic ageing clocks across 13 human tissues, including skeletal muscle, using organ-donor samples, demonstrating heterogeneous ageing trajectories across organs. Together (Table 1), these studies indicate that tissue-resolved proteomes capture ageing signatures that are not fully recapitulated in circulation. While also reinforcing the need for independent biopsy cohorts with functional muscle phenotypes before direct skeletal muscle proteomic clocks can be considered clinically or biologically validated.

Proteomic ageing clocks provide a more direct readout of physiological state than DNAm or mRNA. This functional positioning links proteomic clocks more closely to phenotypic outcomes and disease risk. Proteomic clocks also appear more responsive to perturbations. While DNAm changes can occur rapidly at individual CpG sites but typically require longer periods (weeks to months) to manifest as stable ageing signatures, and transcriptomic responses often peak within hours (e.g. 0–3–6 h following cellular stress), proteomic responses generally occur on an intermediate timescale. Rapid changes in signalling can be detected within hours, whereas changes in protein abundance, particularly for structural, mitochondrial, or ribosomal proteins tend to emerge over hours to days and stabilise over longer periods (days to weeks). Although systematic cross-omic temporal benchmarking remains limited, this intermediate temporal resolution may contribute to the sensitivity of proteomic clocks to interventions such as exercise, weight loss, and pharmacological perturbation [21, 22, 84, 85].

A further practical difference from DNAm clocks is temporal stability and biological proximity to function. DNAm signatures are generally more stable and cumulative, reflecting long-term molecular history and regulatory state [4, 5, 6, 41]. In contrast, protein abundance is more variable and context-dependent, because the proteome represents the functional products of cellular regulation and is directly shaped by inflammation, metabolism, secretion, disease state, medication, exercise, nutrition and sample handling [22, 84, 85]. This context dependence can introduce analytical variability, but it may also make proteomic clocks more responsive to physiological perturbations, whereby changes induced by exercise or therapeutic interventions may manifest more rapidly in the proteome than in the epigenome [86–88]. Collectively, these distinctions highlight the complementary nature of biological-age models but also differences in their objectives. Some highly predictive DNAm clocks are trained on phenotypic and lifestyle variables, meaning they function as risk predictors rather than direct measures of molecular ageing. As multi-omic datasets expand, integrated models combining DNAm, transcriptomic, proteomic, and phenotypic (including wearable-derived) data [89] may outperform single-omic clocks by capturing ageing across multiple temporal and physiological scales.

## Critical appraisal and future directions for proteomic ageing clocks

### Strengths and translational potential

Proteomic ageing clocks offer substantial promise in geroscience due to their close proximity to physiological function. Proteins are a functional layer of cellular biology and therefore reflect the current physiological state, integrating signals from multiple tissues and biological systems. This enables proteomic models to capture pathways directly linked to functional decline and disease risk. Rather than uniformly outperforming other molecular layers, each omic level appears to capture distinct aspects of ageing biology. DNAm reflects cumulative molecular damage, transcriptomics captures dynamic regulatory states, and proteomics provides a readout of functional physiology. As such, proteomic clocks may be particularly informative for conditions characterised by active physiological dysregulation, including inflammation and metabolic dysfunction [1, 17, 90]. This context-dependent sensitivity may explain their strong associations with multimorbidity, frailty, and mortality [21, 50]. Proteomic signatures also demonstrate dynamic responsiveness, making them well suited to intervention studies where molecular changes occur over short timeframes (e.g. exercise, weight loss, pharmacological treatment) [21, 50, 75, 76]. Many of these proteins included in existing clocks are already routinely measured in clinical settings (e.g. cytokines, growth factors, metabolic regulators), supporting their potential integration into existing diagnostic workflows.

### Current limitations and methodological challenges

A central challenge is tissue specificity: most existing models are derived from plasma proteomics, which integrates signals from multiple organs including muscle, liver, adipose tissue and immune cells. While this systemic perspective is valuable, it complicates biological interpretation because circulating proteins represent a composite signal of multiple tissues rather than a direct measure of ageing within any specific organ or cell-type. Conversely, DNAm clocks have greater cross-tissue consistency.

Proteomic ageing clocks are currently built using technologies such as SomaScan, Olink, Alamar, and mass spectrometry, each differing in proteome coverage, dynamic range, and susceptibility to technical artefacts [91], thereby complicating cross-platform comparisons and limiting reproducibility across cohorts. These platforms do not measure identical regions of the proteome. Aptamer-based assays such as SomaScan use modified oligonucleotide binders and can provide broad multiplexed coverage, but measured signals may be influenced by binding affinity, protein isoforms, post-translational modifications and genetic variants affecting aptamer binding [91]. Proximity-extension assays such as Olink use paired antibody-based recognition and DNA barcoding, offering high specificity for predefined panels but coverage that depends on antibody availability and assay design [18, 53, 91]. MS-based proteomics measures peptides directly and can resolve protein isoforms and modification states where coverage is sufficient, but it is constrained by sample complexity, dynamic range and lower sensitivity for very low-abundance proteins [92]. Consequently, the proteins selected into ageing clocks may partly reflect platform coverage and assay chemistry, limiting direct comparison of feature sets across studies without cross-platform benchmarking.

Platform effects are not merely technical details; they can shape the biological interpretation of a clock. Affinity platforms may preferentially capture secreted and clinically assayed proteins, whereas MS-based tissue proteomics may better represent abundant structural, mitochondrial and metabolic proteins [92]. Consequently, a plasma Olink or SomaScan clock and a muscle MS-based clock may select different features because they sample different regions of the proteome, not because one captures ageing biology more accurately than the other [91].

Model interpretability also remains a challenge. While predictive performance is often strong, the mechanistic contribution of individual proteins to ageing pathways is not always clear. In addition, most clocks rely on linear modelling approaches such as elastic net regression. Although robust and interpretable, these methods assume additive relationships and may overlook non-linear interactions or coordinated biological processes. Emerging approaches, including non-linear frameworks such as SNITCH, demonstrate improved capacity to capture complex molecular relationships [93].

Another limitation is the reliance on cross-sectional cohorts, which restricts the ability to quantify rates of biological ageing or determine whether interventions alter ageing trajectories. Many training cohorts lack demographic diversity, with a strong bias towards European ancestry populations. Given that ageing trajectories are shaped by genetic, environmental, and socioeconomic factors, this lack of diversity may reduce model generalisability and introduce bias when applied to underrepresented groups [2, 33].

Finally, proteomic clock outputs (and this applies to outputs of other omic layers) should be interpreted as statistical projections of age-related state onto the proteome. They likely mix deleterious changes with adaptive and compensatory responses, and no single proteomic clock should be taken as a direct and absolute measure of the underlying ageing process. Their value lies in prediction, tissue resolution, responsiveness, and eventual linkage to function, resilience, morbidity, and mortality.

### From systemic ageing signals to tissue and subcellular ageing

A key conceptual development emerging from ageing-clock research is the ability to infer tissue-specific ageing processes from systemic molecular signals. Frameworks such as SystemAge demonstrate that circulating biomarkers can estimate organ-specific ageing from blood measurements [94]. Extending this concept, tissue-resolved proteomic clocks offer the potential to directly quantify ageing within specific organs and subcellular systems [95]. In this framework, ageing is not a single global trajectory but reflects the progressive decline of distinct molecular systems across tissues and subcellular compartments including metabolic, translational, and structural pathways. Proteomic clocks are particularly well suited to capture this resolution because proteins directly represent the functional machinery underlying these pathways.

Recent advances in single-cell, single-fibre and spatial proteomics are beginning to overcome previous technical limitations with tissue-specific ageing clocks. Single-fibre proteomics in human skeletal muscle has revealed fibre-type–specific mitochondrial and metabolic remodelling with ageing and exercise, highlighting substantial cellular heterogeneity masked in bulk tissue analyses [61–63]. Similar approaches across other tissues have identified cell-type–specific proteome remodelling in brain, kidney, liver, and tumour microenvironments [96–99]. Spatial proteomic approaches may further enable mapping of age-associated changes within tissue architecture, including NMJ deterioration and ECM expansion, processes central to age-related functional decline [100–103]. Together, these findings suggest that proteomic ageing trajectories are inherently cell-type dependent and that future ageing clocks may require cellular or spatial resolution to accurately capture tissue ageing processes. Accordingly, these approaches should currently be viewed as complementary to, rather than replacements for, bulk profiling in ageing-clock studies. Single-cell, single-fibre and spatial proteomics can help identify cellular sources, spatial niches and candidate mechanisms of ageing, but clock performance will still require validation in larger bulk tissue and blood datasets with matched phenotypic and functional outcomes.

### Future directions: tissue clocks, multi-omics, and intervention studies

Skeletal muscle represents a particularly compelling target due to its metabolic importance, endocrine function, and early susceptibility to age-related decline [20, 28, 104]. Ageing in skeletal muscle is characterised by coordinated alterations in energy metabolism, protein turnover, and structural organisation, processes that are only partially reflected in circulating proteomic signatures. Although direct skeletal-muscle proteomic age models have recently emerged, they remain limited and lack broad validation across independent biopsy cohorts and muscle-specific functional or longitudinal outcomes, representing a gap not addressed by plasma-derived ageing models. Importantly, skeletal muscle retains substantial plasticity in response to exercise, making it a powerful model for studying plasticity of ageing-associated protein states. Exercise interventions consistently improve mitochondrial function, enhance protein turnover and reduce chronic inflammation [105, 106]. Tissue-specific proteomic clocks may therefore provide a framework to identify which aspects of molecular ageing are modifiable through lifestyle or therapeutic interventions.

The clinical value of muscle-based clocks will depend on their marginal gain over non-invasive blood-based biomarkers. Because muscle biopsies are invasive, direct muscle clocks are unlikely to be first-line diagnostic tools for population screening unless they provide information that cannot be obtained from plasma. Their strongest near-term applications may therefore be in mechanistic studies, intervention trials, validation of blood-based muscle-age proxies, and contexts where biopsies are already collected, such as exercise-training studies, neuromuscular disease research or metabolic phenotyping cohorts. In this framework, biopsy-derived clocks can serve as tissue-anchor models that improve interpretation of less invasive circulating biomarkers. Paired muscle-plasma modelling may therefore provide a practical translational bridge, using biopsy-derived proteomic age as a mechanistic anchor to identify circulating surrogate markers that retain predictive value while being more suitable for repeated or population-scale assessment.

Future models will likely incorporate multiple molecular layers. Integrating proteomics with complementary datasets such as DNA methylation, transcriptomics, and metabolomics may enable ageing clocks that capture both long-term molecular damage and dynamic physiological regulation. Emerging computational frameworks including ensemble models such as EnsembleAge [13], multi-omic integrative factor analysis, and graph-based modelling [65] offer promising strategies for integrating diverse molecular datasets into unified ageing models. Ultimately, the integration of tissue proteomics with circulating biomarkers will be critical for understanding how local molecular ageing processes contribute to systemic biological age. Such frameworks may enable the development of biologically interpretable ageing clocks capable of guiding personalised interventions and improving healthspan. Achieving this integration will require improved standardisation across proteomic platforms, development of longitudinal reference datasets, and clear regulatory frameworks defining how biological-age measures should be applied in clinical and preventive medicine.

## Data Availability

No datasets were generated or analysed during the current study.
